# Phytochemical Profiles, Antimicrobial and Antioxidant Activity of *Knautia integrifolia* (L.) Bertol. subsp. *integrifolia*

**DOI:** 10.3390/plants14030466

**Published:** 2025-02-05

**Authors:** Hilal Kılınc

**Affiliations:** Department of Geological Engineering, Engineering Faculty, Dokuz Eylul University, Izmir 35370, Turkey; hilal.altunkeyik@deu.edu.tr

**Keywords:** *Knautia integrifolia*, Caprifoliaceae, LC-ESI-MS/MS, NMR analysis, antimicrobial activity

## Abstract

The genus *Knautia* (L.) (Caprifoliaceae) is widely distributed in the Mediterranean region and is represented by 11 species of flora in Turkey. This study conducted a detailed phytochemical investigation of the methanol extract of the whole plant of *K. integrifolia* using a combination of LC-ESI-FT-MS and NMR analyses. According to the results of this analysis, 25 compounds were identified in the methanol extract of *K. integrifolia*. The extract is particularly rich in phenolic secondary metabolites, including phenolic acid derivatives, flavonoid glycosides, and flavones, along with the presence of triterpenoid compounds. Additionally, the total phenolic content of the *K. integrifolia* methanol extract was evaluated. Considering the pharmacological activities reported for *Knautia* species, the antioxidant potential of the methanol extract was assessed using the DPPH radical scavenging assay, resulting in a value of 77.5% when compared to the ascorbic acid standard. In this study, antimicrobial activity tests were performed on *K. integrifolia* methanol extract for the first time. The results indicated that the extract demonstrated greater susceptibility to *Staphylococcus epidermidis* compared to the control group. At the same time, *Pseudomonas aeruginosa* exhibited a minimum inhibitory concentration value, indicating high sensitivity to the methanol extract.

## 1. Introduction

*Knautia integrifolia* (L.) Bertol. subsp. *integrifolia* is a plant belonging to the Caprifoliaceae family and is characterized as an annual plant with lavender−blue or pinkish flowers [1]. According to the results of the Angiosperm Phylogeny Group’s new molecular phylogenetic and morphological research study, *Knautia* has been included in the Caprifoliaceae family and has moved from the Dipsacaceae family [2,3].

The genus encompasses approximately 60 species that have been identified, especially in the Mediterranean Basin and in Turkey, Southern Europe, the Alps, the Balkans, the Aegean Islands, and with 11 species occurring in the flora of Turkey [4].

Extracts from various species of *Knautia* have been used in traditional medicine due to their antioxidant, antimicrobial, antiproteolytic, anti-inflammatory, expectorant, diuretic, analgesic, and elastase inhibitory activities [5,6,7]. While the potential medicinal properties of the *Knautia* genus have not been extensively researched, some studies are available on its uses. *K. arvensis* is used as a remedy for some skin disorders, and tea made from its flowers and leaves is used for some lung problems [8]. It has also found applications as a muscle relaxant and blood purifier [9], as well as in agriculture as an alternative to antibiotic growth promoters in animal feed due to its potential antiproteolytic activity [10].

Extracts of *Knautia drymeia* and *Knautia macedonica* are used to treat inflammation-related disorders, including acne [11]. In particular, ethanol and methanol–acetone–water extracts obtained from the aerial parts of *K. drymeia* exhibited strong antioxidant, antibacterial, and anti-inflammatory effects. In a recent study, the high binding affinity demonstrated by *Knautia sarajevensis* against the main protease of SARS-CoV-2 virus indicated the potential therapeutic use of this plant [12].

Literature studies have shown that the *Knautia* species contain many important bioactive constituents, including triterpene saponins [13], flavonoids [14,15], phenolic acids, flavone glycosides [16], phenol carboxylic acids, sugar derivatives, phytosterols, fatty acids, and their esters, and volatile compounds [11].

This study aimed to provide a comprehensive phytochemical characterization of the whole *K. integrifolia* plant extract through integrated LC-ESI-MS/MS and 1D- and 2D-NMR analyses. Phytochemical investigation revealed that *K. integrifolia* contains natural compounds belonging to the terpenoid, flavonoid, and phenolic acid classes, and their structures were elucidated. Additionally, the antioxidant and antimicrobial activities and the minimum inhibitory concentration (MIC) of the methanol extract of *K. integrifolia* were evaluated.

## 2. Results and Discussion

### 2.1. LC-ESI-MS/MS Analysis of Knautia integrifolia Methanol Extract

**Figure 1:**
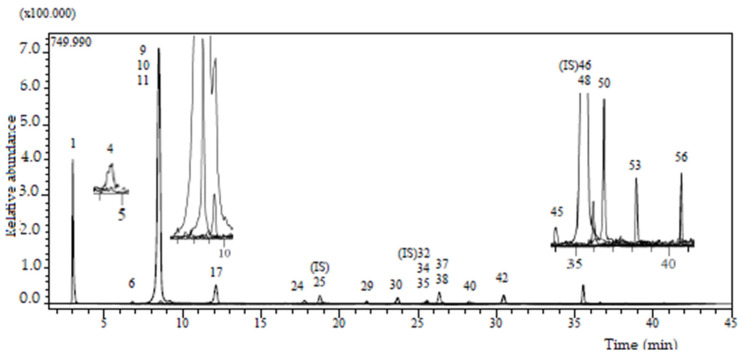
LC-ESI-MS/MS profile (base peak chromatogram) of *Knautia integrifolia* methanolic extract.

**Figure 2:**
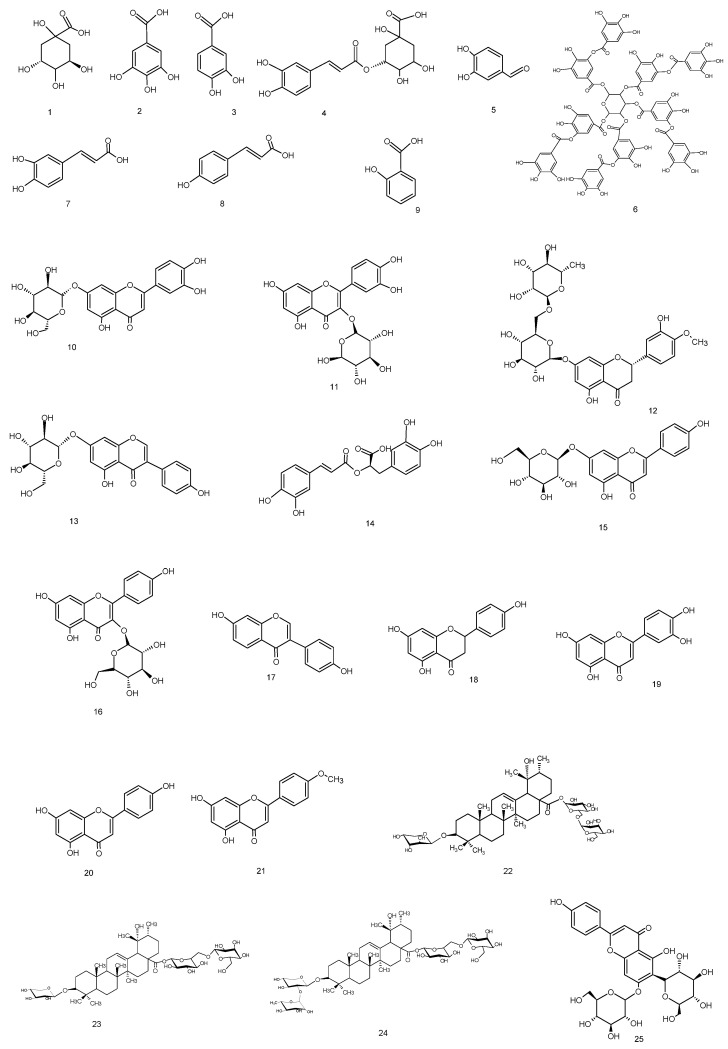
Compounds occurring in *Knautia integrifolia* methanol extract.

The primary objective of this study was to investigate the metabolites present in *Knautia integrifolia* through a comprehensive phytochemical investigation. To accomplish this, meticulous analysis was conducted to thoroughly understand the constituents of the methanol extract of *K. integrifolia* and precisely identify the peaks observed in the LC-ESI-MS-MS profile (Figure 1). Utilizing UHPLC-MS/MS, phenolic substances were detected in the extract. The screening involved 53 phenolic compounds, with their respective concentrations expressed in milligrams per gram of extract; 21 fingerprint phytochemical compounds were identified and quantified, as shown in Table 1 (Figure 2).

The analysis confirmed that the methanolic extract contained five phenolic acids, four flavonoid glycosides, four flavonoid aglycones (flavones), two caffeic acid esters, an organic acid, a phenolic aldehyde, a polyphenol, an isoflavone, an isoflavone glycoside, and a flavanone glycoside. It was determined that the extract contained the highest quantities of quinic acid (8.094 mg/g) and chlorogenic acid (4.749 mg/g) among the 53 standard compounds. The other components were present in medium to low amounts. Chlorogenic acid is produced by the esterification of caffeic acid and quinic acid.

In contrast, quinic acid is derived from the hydrolysis of chlorogenic acid, and a strong relationship exists between these two compounds. The therapeutic effects of quinic acid and chlorogenic acid were investigated, and they were found to have antioxidant and antibacterial properties. In this study, antimicrobial and antioxidant activity tests were applied to the methanol extract of *K. integrifolia*, and it was observed that both activities were significant [17].

A comparison of the obtained results with literature data showed that several phenolic compounds, such as gallic acid, salicylic acid, 4-hydroxybenzoic acid, vanillic acid, chlorogenic acid, caffeic acid, sinapic acid, ferulic acid, rosmarinic acid, p-coumaric acid, myricetin, kaempferol, and apigenin, have already been reported in the *Knautia* genus [12,18,19,20]. In addition, a study on *K. integrifolia* (L.) Bertol. found the presence of flavonoids such as cynaroside, luteolin, and cosmosiin [14].

According to the LC-ESI-MS/MS analysis results, the methanol extract of *K. integrifolia* is rich in valuable phenolic secondary metabolites, mainly phenolic acid derivatives, flavonoid glycosides, and flavones. In addition, compounds not previously reported in the literature for this species were detected. This includes compounds **1**, **3**, **5**, **6**, **11**–**13**, and **21** within the methanolic extract of *Knautia integrifolia* (L.) Bertol. subsp. *integrifolia*. A comprehensive phytochemical analysis of the methanol extract was conducted with the aim of obtaining a thorough understanding of its constituents and accurately attributing the peaks observed in the LC-ESI-MS/MS profile.

### 2.2. Isolation and Characterization of Specialized Metabolites from Knautia integrifolia

**Figure 3:**
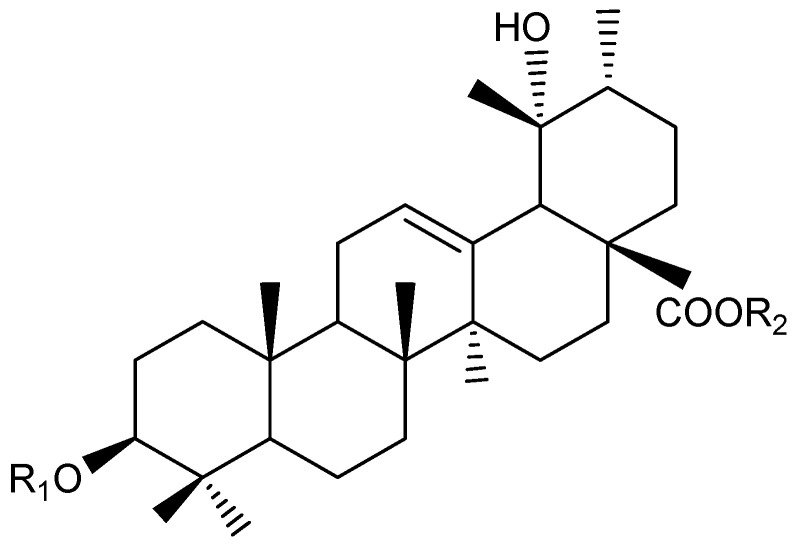
Ursane-type saponins structure (22, 23, 24) from *Knautia integrifolia*. Compound **22**: R_1_ = Ara, R_2_ = All (1 → 6) Glc, Compound **23**: R_1_ = Xyl, R_2_ = All (1 → 6) Glc, Compound **24**: R_1_ = Rha (1 → 2) Ara, R_2_ = All (1 → 6) Glc.

The methanolic extract of *Knautia integrifolia* was partitioned between n-butanol and water, and the n-butanol extract was subjected to size exclusion chromatography using Sephadex LH-20 for the first separation. Open-column chromatography was used for further purification, leading to the isolation of phenolic and triterpene compounds. Some of the phenolic compounds **1**–**4**, **7**, **10**, **14**, and **15** listed in the LC-ESI-MS/MS table were isolated from the methanolic extract of *K. integrifolia*, and their structures were identified and are shown in Table 1 (Figure 2). Additionally, terpenoid and phenolic compounds, including **22** (Bidenoside A), **23** (Scabrioside A), **24** (Scabrioside C), and **25** (Saponarin), which are not listed in the table, were also isolated, and their structures were thoroughly characterized (Figure 2 and Figure 3). Their structures were elucidated by extensive spectroscopic methods, including 1D- (1H) and 2D-NMR (DQF-COSY, HSQC, and HMBC) experiments, as well as LC-ESI-MS/MS analysis. All triterpene metabolites isolated and elucidated by NMR are reported in Table 2 and Table 3.

Through meticulous analysis of the NMR data, the triterpene-type compounds isolated and purified from the methanolic extract of *K. integrifolia* using a combination chromatographic method were found to share the same aglycone. However, they were characterized by different sugar chain combinations linked to C-3 and C-28. Compounds **22**, **23,** and **24** were identified as ursane-type triterpenes based on ^1^H NMR and ^13^C NMR analyses [21,22].

The positive HR-MS spectrum of compound **22** was characterized by an [M + H]^+^ peak at *m*/*z* 929.5020, corresponding to a molecular formula of C_47_H_76_O_18_. The ^13^C NMR spectrum showed 47 carbon signals, of which 30 were assigned to the aglycone moiety and 17 to a sugar portion made up of three sugar units. The ^1^H NMR spectrum displayed signals for six tertiary methyl groups at δ 0.80, 0.88, 0.95, 1.07, 1.22, and 1.33, a secondary methyl group at δ 0.96 (d, *J* = 6.7 Hz), an olefinic proton at δ 5.33, and one oxygen-bearing methine proton at δ 3.16 (dd, *J* = 11.5, 4.5 Hz, H-3) (Table 2 and Table 3). Additionally, three anomeric proton signals at δ = 4.32 (d, *J* = 6.8 Hz), 4.71 (d, *J* = 7.5 Hz), and 5.31 (d, *J* = 8.0 Hz), which were assigned to one β-glucopyranosyl (δ 5.31), one β-allopyranosyl (4.71) and one α-arabinopyranosyl (δ 4.32) units. These signals, along with the carbon resonances in the ^13^C NMR spectrum for a carboxyl group at δ 178.5, two unsaturated carbons at δ 138.4 and 129.4, two hydroxyl-substituted carbons at δ 90.6 and 72.8, and seven methyl groups at δ 16.2, 16.3, 16.5, 16.8, 24.3, 26.8, and 27.5, indicated that the aglycone of compound **22** was a 19-oxygenated urs-12-en type triterpene saponin, pomolic acid derivative [23], already reported in *K. integrifolia* [13]. Thus, the structure of compound **22** was established as 3-O-α-L-arabinopyranosyl-28-O- [β-D-allopyranosyl (1 → 6)-β-D-glucopyranosyl]-pomolic acid and named bidenoside A.

The positive HR-MS spectrum of compound **23** was characterized by an [M + Na]^+^ peak at *m*/*z* 951.4922, corresponding to a molecular formula of C_47_H_76_O_18_. The ^13^C NMR analysis of compound **23** displayed 47 carbon signals. Among these, 17 were attributed to a pentose and two hexose units, while the remaining 30 signals were associated with a triterpenoid skeleton. The ^13^C NMR spectrum showed signals of a pair of olefinic carbons at δ 128.0 and 138.4, three anomeric carbons at δ 94.5, 101.2, and 105.6, and a carbonyl carbon at δ 178.5. A comparison of the signals from the aglycone of **23** in the ^13^C NMR spectrum with the literature showed that the aglycone of **23** was pomolic acid (3β,19α-dihydroxyurs-12-ene-28-oic acid) [23]. The spin systems of the sugars were assigned based on spectroscopic evidence obtained by DQF-COSY and HSQC experiments. The sugar linkages were determined by the HMBC spectrum. The ^1^H NMR spectrum displayed signals for the anomeric protons of sugar units at δ 4.31 (d, *J* = 7.4 Hz), 5.31 (d, *J* = 7.5 Hz), and 4.69 (d, *J* = 8 Hz), corresponding to the anomeric protons of D-xylose, D-glucose, and D-allose, respectively (Table 2 and Table 3). The HMBC correlations between the proton signal at δ 5.31 (H-1_glc_) and a carbonyl carbon signal at δ 178.5 (C-28), whereas a proton signal at δ 4.69 (H-1_all_) correlated with the carbon signal at (C-6_glc_). A proton signal at δ 4.31 (H-1_ara_) had an across peak with a carbon signal at 90.2 (C-3) in the HMBC spectrum. All this evidence indicated that the two sugar moieties of **23** were linked at both C-3 and C-28 of oleanolic acid. Therefore, the structure of compound **23** was established as 3-O-β-D-xylopyranosyl-28-O-β-D-allopyranosyl-(1 → 6)-β-D-glucopyranosyl-pomolic acid and named scabrioside A.

The positive HR-MS spectrum of compound **24** was characterized by an [M + Na]^+^ peak at *m*/*z* 1097.4929, corresponding to a molecular formula of C_53_H_86_O_22_. According to the ^13^C NMR spectrum, it was observed that the structure consists of 53 carbons, 30 of which belong to the aglycone moiety and 23 to a sugar portion made up of four sugar units (Table 2 and Table 3). A pair of signals at δ 128.9 (C-12) and δ 138.5 (C-13) were indicative of the double bond in an urs-12-ene type structure. Additionally, carbon signals at δ 90.2 (C-3) and δ 73.1 (C-19) corresponded to hydroxylated carbons. Lastly, a singlet proton signal at δ 2.53, and the corresponding carbon resonance at δ 53.9, were assigned to H-18 and C-18, respectively. These results confirmed the presence of pomolic acid (3β,19α-dihydroxyurs-12-ene-28-oic acid) as the aglycone of compound **24**. The ^1^H and ^13^C NMR spectra of compound **24** showed many similarities to those of compound **22**, especially for the signals attributed to pomolic acid, α-L-arabinose, β-D-glucose, and β-D-allose (Table 2 and Table 3). However, differences were observed in the anomeric proton signal (δ 5.11), and the corresponding carbon signals were identified as α-L-rhamnose through COSY and HMBC experiments. The HMBC correlations between the proton signal at δ 5.30 (H-1_glc_) and a C-28 carbon signal at δ 179.0 and C-6_glc_ (δ 67.9) of the β-D -glucose and H-1_all_ (δ 4.70) of the terminal β-D-allose unit were observed. The attachment of the α-L-arabinose unit to the C-3 position of the aglycone was confirmed by a COSY experiment, and a strong interaction was observed between the H-3 proton of the aglycone (δ 3.15) and the H-1_ara_ (δ 4.56) proton of α-L-arabinose. In addition, the HMBC experiment performed with compound **24** revealed long-range correlations between the C-2_ara_ carbonyl of α-L-arabinose (δ 76.1) and the H-1_rha_ proton of α-L-rhamnose (δ 5.11) [23]. Thus, the structure of compound **24** was established as 3-O-α-L-rhamnopyranosyl-(1 → 2)-α-L-arabinopyranosyl-(-28-O-β-D-allopyranosyl-(1 → 6)-β-D-glucopyranosyl-pomolic acid and named scabrioside C.

### 2.3. Evaluation of the Antioxidant Activity

**Figure 4:**
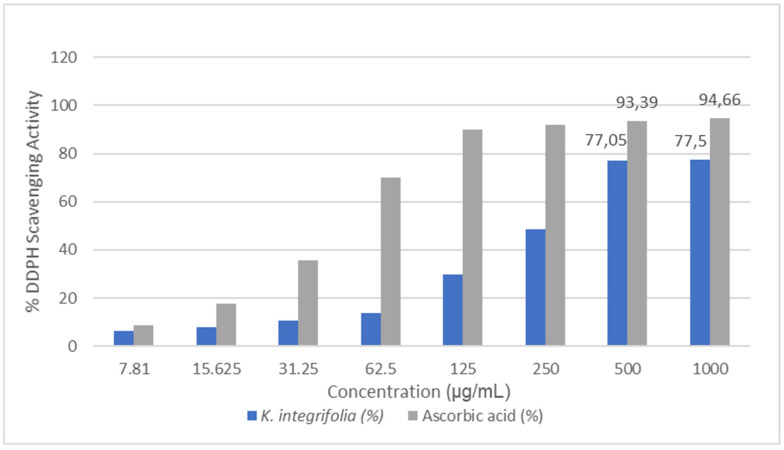
DPPH radical scavenging activity results for *Knautia integrifolia* and ascorbic acid (%).

This research evaluated the antioxidant potential of a methanol extract from *Knautia integrifolia* through a DPPH radical scavenging assay. Different extract concentrations ranging from 7.81 µg/mL to 1000 µg/mL were tested. The results of the DPPH radical scavenging activity for both *K. integrifolia* and ascorbic acid are summarized in Figure 4. The antioxidant activity of the methanol extract obtained from *K. integrifolia* was compared to that of the control group, ascorbic acid. The *K. integrifolia* methanol extract showed inhibition rates of 77.5% and 75.07% at concentrations of 1000 µg/mL and 500 µg/mL, respectively. The methanol extract of *K. integrifolia* is rich in various phenolic compounds, including chlorogenic acid, quinic acid, hesperidin, saponarin, and astragalin, which are well-known for their food preservation properties and potent antioxidant activities [17,24,25]. These findings classify *K. integrifolia* as a plant abundant in natural antioxidants and underscore the potential of *Knautia* species as a valuable source of natural antioxidants. Furthermore, the results obtained from this study were compared with data from the literature, revealing that *Knautia arvensis*, *Knautia drymeia*, and *Knautia sarajevensis* species exhibit strong activities against DPPH radicals [11,19,26,27]. It has been determined that these results are consistent with the literature data.

### 2.4. Antimicrobial Activity of Knautia integrifolia

**Figure 5:**
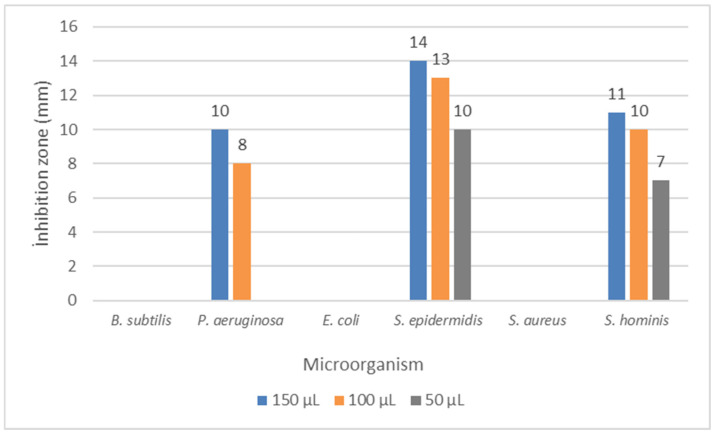
Disc diffusion results for *Knautia integrifolia*. *B. subtilis*: *Bacillus subtilis*, *P. aeruginosa*: *Pseudomonas aeruginosa*, *E. coli*: *Escherichia coli*, *S. epidermidis*: *Staphylococcus epidermidis*, *S. aureus*: *Staphylococcus aureus*, and *S. hominis*: *Staphylococcus hominis*.

The present study investigated the antimicrobial activities of the methanol extract of *Knautia integrifolia* against various microorganisms, both qualitatively and quantitatively, through the examination of inhibition zones and minimum inhibitory concentration (MIC). The methanolic extract exhibited moderate antimicrobial effects against the tested microorganisms, with inhibition zones ranging from 8 to 14 mm, as shown in Figure 5. Remarkably, *Staphylococcus epidermidis* demonstrated higher susceptibility to the plant extract compared to an empty ethanol-loaded disk, displaying an inhibition zone of 14 mm. Likewise, *Staphylococcus hominis* and *Pseudomonas aeruginosa* exhibited inhibition zones of 11 mm and 10 mm, respectively. It was observed that at the tested concentrations, there was no effect against *Bacillus subtilis*, *Escherichia coli*, and *Staphylococcus aureus*.

According to the results of the minimum inhibition concentration (MIC) values, *P. aeruginosa* displayed sensitivity to the extract among the tested bacteria, with an MIC value of 260 µg/mL. This was followed by *Pseudomonas fluorescens*, which demonstrated moderate activity with an MIC value of 2080 µg/mL. *Staphylococcus hominis* 4170 µg/mL, *Salmonella typhimurium* 16,700 µg/mL, and *Staphylococcus epidermidis* 16,700 µg/mL exhibited low sensitivity. The extract’s strong activity against *P. aeruginosa*, a highly versatile and pathogenic bacterium, is particularly significant, as this organism poses a severe threat by causing persistent infections in burn patients, immunocompromised individuals, and those with cystic fibrosis [28].

Plants from the Caprifoliaceae family, to which *Knautia* belongs, are rich in bioactive compounds, such as flavonoids [19] and saponins [29], which are likely responsible for their observed biological activities. Although there are limited reports on the chemical composition and biological activity of *Knautia* species, studies have indicated that their methanol extracts contain phenolic acids, which may contribute to their antimicrobial properties. Previous research has demonstrated the antimicrobial activity of methanol extracts against various bacterial strains [11,20].

The antibacterial effects of terpenoids and flavonoids are likely attributed to the disruption of microbial membranes by terpenes, as well as the ability of flavones to form complexes with extracellular and soluble proteins and bacterial cell walls, thereby compromising membrane integrity [30].

Expanding on these observations, when the methanol extract of the plant was analyzed, it revealed the presence of not only phenolic compounds but also pomolic acid, a pentacyclic triterpenoid of the ursane type, which is well-known for its anticancer properties [31]. The phenolic compounds in the extract also demonstrate bacteriostatic or bactericidal activity against both Gram-positive and Gram-negative bacteria, highlighting their potential as a promising source for the development of antimicrobial agents. Their functional groups allow for diverse mechanisms of action, including membrane disruption and enzyme inhibition [32]. The antimicrobial properties of biologically active secondary metabolites are influenced by factors such as the structural differences in bacterial cells, concentration of secondary metabolites in the extract, type of metabolites, extraction method, and exposure time of bacteria to the metabolites [33]. Based on all these findings, the activity of the methanol extract against the tested bacteria can be attributed to the bioactive secondary metabolites present in its composition.

This study highlights the notable antimicrobial activity of *K. integrifolia*, despite the scarcity of prior research on this species. By demonstrating its potential as a source of bioactive compounds, this research provides a foundation for further exploration of *Knautia* species and their potential application in drug development.

## 3. Materials and Methods

### 3.1. General Procedures

NMR experiments were carried out using methanol-*d*4 (99.95%, Sigma-Aldrich, Milan, Italy) as the solvent on a Bruker DRX-600 spectrometer (Bruker BioSpin GmBH, Rheinstetten, Germany) equipped with a Bruker 5 mm TCI CryoProbe at 300 K. Data were analyzed using Topspin 3.2 software, and ROESY spectra were acquired with tmix = 400 ms.

The quantitative evaluation of phytochemicals was performed using a Shimadzu-Nexera model ultrahigh performance liquid chromatography (UHPLC) coupled with a tandem mass spectrometer. Mass spectrometric detection was conducted using a Shimadzu LCMS-8040 model tandem mass spectrometer operating proficiently in both negative and positive ionization modes via electrospray ionization (ESI).

TLC was performed on silica gel F_254_ (Merck 5554) plates (20 cm × 10 cm), and mixtures of CH_2_Cl_2_–CH_3_OH–H_2_O (80:20:2, 70:30:3, and 61:32:7) were used as mobile phases to obtain a separation distance of 80 mm. For thin-layer chromatography, silica gel F_254_ (Merck 5554) and RP-18 F_254s_ (Merck 5560) precoated plates were used. Detection was carried out by spraying 20% H_2_SO_4,_ followed by heating at 105 °C for 5 min. Silica gel 60 (0.063–0.200 mm, Merck), Sephadex LH 20 (25–100 μm, Sigma-Aldrich), and LiChroprep RP-18 (25–40 μm, Merck) were used for open-column chromatography. Silica gel 60 (0.063–0.200 mm, Merck) was used for column chromatography. Biotek Microplate spectrophotometer was used for spectrophotometric assays.

### 3.2. Plant Material

*Knautia integrifolia* (L.) Bertol. subsp. *integrifolia* (whole plant) was collected in Bornova (GPS coordinates latitude: 38.27483, longitude: 27.14120), Izmir, Turkey, in May 2022. A voucher specimen was deposited in the Herbarium of the Science Faculty, Ege University, Izmir, Turkey (EGE 43851).

### 3.3. LC-ESI-MS/MS Analysis

The methanol extract of *Knautia integrifolia* was subjected to analysis using ultra-high-performance liquid chromatography (UHPLC) in conjunction with Tandem Mass Spectrometry, employing the method previously described and validated by Yilmaz et al.; 2020 [34]. Chromatographic parameters were systematically optimized to achieve exceptional separation efficiency for 53 phytochemical constituents and to overcome the suppression effects. All experiments were carried out using an autosampler (SIL-30AC model), a column oven (CTO-10ASvp model), binary pumps (LC-30AD model), and a degasser (DGU- 20A3R model). A comprehensive exploration involving a variety of chromatographic columns, such as the Agilent Poroshell 120 EC-C18 model (150 mm × 2.1 mm, 2.7 µm) and RP-C18 Inertsil ODS-4 (100 mm × 2,1 mm, 2 µm), as well as different mobile phases (B) such as acetonitrile and methanol, and mobile phase additives such as ammonium formate, formic acid, ammonium acetate, and acetic acid, was conducted. Additionally, different column temperatures ranging from 25 °C to 40 °C were tested and optimized until the optimum conditions were achieved. Subsequently, chromatographic separation was performed on a reversed-phase Agilent Poroshell 120 EC-C18 model (150 mm × 2.1 mm, 2.7 µm) analytical column, with the column temperature set at 40 °C. The elution gradient was composed of eluent A (water + 5 mM ammonium formate + 0.1% formic acid) and eluent B (methanol + 5 mM ammonium formate + 0.1% formic acid). The gradient profile was programmed as follows: 20–100% B over 0–25 min, 100% B maintained from 25 to 35 min, and a return to 20% B from 35 to 45 min. The solvent flow rate was set at 0.5 mL/min, and the injection volume was fixed at 5 µL.

Mass spectrometric detection was performed using a Shimadzu LCMS-8040 model tandem mass spectrometer with an electrospray ionization (ESI) source operating in both negative and positive ionization modes. LabSolutions software (Shimadzu) was used for data acquisition and processing. The multiple reaction monitoring (MRM) mode was utilized for phytochemical quantification, with parameters optimized for precise precursor-to-fragment ion transitions. Collision energies (CE) were adjusted to maximize fragmentation efficiency and ensure effective transmission of the desired product ions. The operating conditions for the mass spectrometer included a drying gas (N_2_) flow of 15 L/min, nebulizing gas (N_2_) flow of 3 L/min, DL temperature of 250 °C, heat block temperature of 400 °C, and interface temperature of 350 °C.

### 3.4. Extraction and Isolation Procedure

The whole plant material (1291 g), air-dried and powdered, was extracted with hexane (2 × 2.5 L), dichloromethane (2 × 2.5 L), and methanol (3 × 2.5 L) at room temperature. After filtration and solvent evaporation under vacuum, 142.6 g of a light-brown residue was obtained. The residue was dissolved in water and then partitioned with n-butanol saturated with H_2_O (2 × 400 mL). The n-butanol extract (64.1 g) was obtained after filtration and evaporation of the solvent to dryness under reduced pressure. A portion of the n-butanol extract (7.5 g) was subsequently fractionated on a Sephadex column, and 7.5 g of n-butanol extract was fractionated on a Sephadex.

LH-20 column (100 × 5 cm), using MeOH as the mobile phase, affording 84 main fractions (10 mL), as monitored by TLC. Fractions with similar Rf values were combined to yield six major fractions (A–F). Further purification was carried out by open-column chromatography with CH_2_CI_2_-MeOH-H_2_O at different percentages.

Fraction B (1150 mg) was further applied to open CC (350 g) using CH_2_CI_2_-MeOH-H_2_O (80:20:2, 2000 mL; 70:30:3, 1000 mL and 61:32:7, 1500 mL) as eluent to give 16 subfractions. Subfraction 1 (11 mg) was purified on a Sephadex column (10 g) using MeOH (100 mL) as the mobile phase to afford compound **2** (1.9 mg). Subfraction 7 (20 mg) was submitted to a Si gel column (10 g) with the solvent system CH_2_CI_2_-MeOH mixtures (9:1, 50 mL and 8:2, 50 mL) to yield compounds **22** (1.9 mg) and **10** (0.8 mg). Subfraction 8 (235 mg) was further purified on a normal phase silica gel column (50 g) employing MeOH–H_2_O (9:1, 50 mL; 8:2, 50 mL; and 7:3, 50 mL) to give compound **23** (0.6 mg). Subfraction 10 (36 mg) was applied to a silica gel column (12 g) using MeOH–H_2_O (9:1, 50 mL; 8:2, 50 mL) to obtain compound **24** (5.5 mg). Subfraction 14 (32 mg) was further purified on a normal phase silica gel column (15 g) employing MeOH–H_2_O (9:1, 50 mL) to give compound **1** (8.2 mg).

Fraction E (194 mg) was further separated over a normal phase silica gel column (90 g) with the solvent system CH_2_CI_2_-MeOH-H_2_O mixtures (70:30:3, 1000 mL) to afford compounds **25** (25.3 mg), **14** (0.9 mg), **15** (1.7 mg), and **4** (2.3 mg).

Fraction F (501 mg) was fractionated over an open CC using silica gel (200 g) as a stationary phase. Elution was performed with CH_2_CI_2_-MeOH-H_2_O (70:30:3, 500 mL; 61:32:7, 1000 mL) to yield compound **7** (1.8 mg).

Fraction G (587 mg) was applied to a silica gel column (200 g) using CH_2_CI_2_-MeOH-H_2_O (70:30:3, 1000 mL and 61:32:7, 1500 mL) as the eluent to give compound **3** (8.25 mg).

### 3.5. Determination of Antioxidant Activity

The antioxidant potential of the methanol extract was evaluated using the stable 2,2-diphenyl-1-picrylhydrazyl radical (DPPH•) following the procedure previously described [35]. To prepare the DPPH• solution, 3.9432 mg of DPPH (2,2-diphenyl-1-picrylhydrazyl) was weighed on a precision balance and added to 50 mL of ethanol. A glass bottle containing the DPPH• solution was covered with aluminum foil to protect it from light. The extract was mixed with DPPH• solution and incubated at room temperature for 30 min in the dark [36]. Ascorbic acid served as the positive control, while methanol was used as the negative control. After incubation, the absorbance was measured at 515 nm using a UV–visible spectrophotometer (Biotek Microplate spectrophotometer, ABD) at 515 nm. All experiments were conducted in triplicate. Radical scavenging activity was expressed as percentage inhibition of DPPH• radical and was calculated by the following equation:

% Inhibition = [(A_control_ − A_sample_)/A_control_] × 100 (1)

### 3.6. Antimicrobial Activity Assays

Test bacteria strains used in the study were *Bacillus subtilis* DSMZ 1971, *Escherichia coli* ATCC 25922, *Pseudomonas aeruginosa* DSMZ 50071, *Staphylococcus aureus* ATCC 25923, *Staphylococcus epidermidis* DSMZ 20044, *Pseudomonas fluorescens* P1, *Salmonella typhimurium* SL 1344, and *Staphylococcus hominis* ATCC 27844. The microorganism inocula were prepared under controlled conditions of correct incubation, with bacteria undergoing a 24-h incubation period at 37 °C, while yeasts were subjected to 48 h of incubation at 27 °C. Sterile saline solutions were carefully prepared for each microorganism, ensuring purity and consistency, and subsequently adjusted to the 0.5 McFarland standard to obtain uniformity in cell density across samples. Prepared according to the turbidity of the 0.5 McFarland standard, the concentration of viable cells within the test tube ranged from 1 × 107 to 1 × 108 colony-forming units per milliliter (cfu/mL) [37]. The disk diffusion test, a widely employed method, was used to evaluate antimicrobial activity [38]. The extract was applied onto 6 mm Oxoid antimicrobial susceptibility test disks at three doses: 50, 100, and 150 μL each. After application, the disks were air-dried for 8 h at 30 °C under sterile conditions to remove residual ethanol from the extract. Autoclaved, sterilized Mueller−Hinton agar (BD Difco, Franklin Lakes, NJ, USA) was poured into 90 mm sterile Petri dishes to achieve an average depth of 4.0 mm (±0.5 mm). Microorganism suspensions were evenly spread across the surface of the Petri dishes. Inside the biosafety cabinet, Petri dishes containing microorganism suspensions were allowed to air-dry for 5 min before disk placement. Disks were positioned on the agar surface within 10 min. Following completion of the test, Petri dishes were incubated at 37 °C for 24 h, and the diameters of the inhibition zones were measured in mm. Control discs, sterile and impregnated with ethanol, were utilized as negative controls in this study [35]. Prior to implementation, these ethanol-laden discs underwent exact drying under aseptic conditions to ensure the complete removal of ethanol residues.

Minimum inhibitory concentration (MIC) values of the extracts were determined using the micro-well dilution method against sensitive bacteria in the agar-well diffusion experiment. For the MIC method, microplates were filled with Mueller−Hinton broth (liquid medium). Initially, 100 µL of the extract was added to the wells of the first column, followed by serial dilution. Then, 50 µL of standardized microorganisms were placed in the microplate containing the diluted extracts. The loaded microplates were then incubated at 37 °C for 18 h. Wells devoid of microorganism challenge were used as negative controls [39]. The initial concentration without visible growth was determined as the MIC (µg/mL).

## 4. Conclusions

This study focused on the extraction and isolation of secondary metabolites from the methanol extract of the whole plant *Knautia integrifolia*, a species of Turkish flora, as well as the elucidation of their structures through a combination of comprehensive LC-ESI-MS/MS and NMR analyses. According to the results of detailed and in-depth analyses with precise measurements of the methanol extract of *K. integrifolia*, 25 secondary metabolites were identified, including phenolic acid derivatives, flavonoid glycosides, and flavones, along with triterpenoid compounds.

Among these compounds, quinic acid, protocatechuic acid, protocatechuic aldehyde, tannic acid, isoquercitrin, hesperidin, genistin, acacetin, saponarin, scabrioside A, and scabrioside C were identified for the first time in this plant.

The tested extract exhibited good antioxidant activity and demonstrated a notable effect in scavenging free radicals. These results highlight the significant role of secondary metabolites in establishing a natural antioxidant source. In addition, when the antimicrobial activity test results were examined, *Staphylococcus epidermidis* showed significantly higher sensitivity to the plant extract. Furthermore, the methanol extract of *K. integrifolia* displayed particularly high MIC values against *P. aeruginosa*. To our knowledge, this is the first study to investigate the antimicrobial activity of this plant species.

These results highlight the potential of this plant species as a source of bioactive molecules with therapeutic properties, particularly as antimicrobial and antioxidant agents. These findings underscore its value for developing new pharmaceutical products and biomedical applications. However, further in vitro and in vivo clinical studies are essential to clarify the mechanisms of action and evaluate the safety profile of the tested extracts.

Future research could explore the synergistic effects of combining the different secondary metabolites identified in this study, which may lead to enhanced therapeutic outcomes. Additionally, studies on the bioavailability, pharmacokinetics, and potential toxicity of the identified compounds will provide deeper insights into their suitability for clinical applications.

This research focused on a comprehensive phytochemical analysis of the methanol extract of *K. integrifolia*. Although limited to a single species, this study was conducted within a rigorous and reproducible framework, serving as a valuable reference for researchers interested in exploring other *Knautia* species. Further studies on other species and isolated compounds could help uncover the potential applications of these plants in the pharmaceutical, food, or cosmetic industries.

Furthermore, the sustainable extraction and utilization of bioactive compounds from *K. integrifolia* could contribute to the growing field of natural product chemistry, offering eco-friendly alternatives to synthetic chemicals in various industries.

## Figures and Tables

**Table 1:** Compounds identified in the *Knautia integrifolia* extract by LC-ESI-MS/MS analysis.

Numbers	Analytes	RT	M.I. (*m*/*z*)	F.I. (*m*/*z*)	Ion. Mode	mg Analyte/g Extract
1	Quinic acid	3.0	190.8	93.0	Neg	8.094
2	Gallic acid	4.4	168.8	79.0	Neg	0.005
3	Protocatechuic acid	6.8	152.8	108.0	Neg	0.057
4	Chlorogenic acid	8.4	353.0	85.0	Neg	4.749
5	Protocatechuic aldehyde	8.5	137.2	92.0	Neg	0.051
6	Tannic acid	9.2	182.8	78.0	Neg	0.009
7	Caffeic acid	12.1	179.0	134.0	Neg	0.099
8	p-Coumaric acid	17.8	163.0	93.0	Neg	0.018
9	Salicylic acid	21.8	137.2	65.0	Neg	0.011
10	Cyranoside	23.7	447.0	284.0	Neg	0.023
11	Isoquercitrin	25.6	463.0	271.0	Neg	0.137
12	Hesperidin	25.8	611.2	449.0	Pos	0.002
13	Genistin	26.3	431.0	239.0	Neg	0.185
14	Rosmarinic acid	26.6	359.0	197.0	Neg	0.005
15	Cosmosiin	28.2	431.0	269.0	Neg	0.191
16	Astragalin	30.4	447.0	255.0	Neg	0.288
17	Daidzein	34.0	253.0	223.0	Neg	0.002
18	Naringenin	35.9	270.9	119.0	Neg	0.002
19	Luteolin	36.7	284.8	151.0/175.0	Neg	0.003
20	Apigenin	38.2	268.8	151.0/149.0	Neg	0.001
21	Acacetin	40.7	283.0	239.0	Neg	0.002

RT: Retention time, M.I. (*m*/*z*): Molecular ions of the standard analytes (*m*/*z* ratio), F.I. (*m*/*z*): Fragment ions.

**Table 2:** ^13^C and ^1^H NMR data (*J* in Hz) of the aglycone moieties of compounds **22**, **23**, and **24** (150 and 600 MHz, δ ppm, in methanol-*d*4).

	22		23		24	
	δ_C_	δ_H_ (*J* in Hz)	δ_C_	δ_H_ (*J* in Hz)	δ_C_	δ_H_ (*J* in Hz)
1	39.4	1.67, 1.02, *m*	39.1	1.66, 1.02, *m*	39.1	1.67, 1.03, *m*
2	26.8	1.86, 1.65, *m*	26.9	1.86, 1.67, *m*	26.8	1.85, 1.66, *m*
3	90.6	3.17, dd (11.5, 4.6)	90.2	3.17, dd (11.5, 4.6)	90.2	3.15, dd (11.5, 4.6)
4	39.7	-	40.1	-	39.3	-
5	56.5	0.81, *m*	56.1	0.80, *m*	56.6	0.80, *m*
6	19.0	1.55, 1.42, *m*	18.1	1.56, 1.41, *m*	18.1	1.55, 1.42, *m*
7	33.5	1.56, 1.34, *m*	32.6	1.54, 1.33, *m*	32.6	1.56, 1.32, *m*
8	40.4	-	40.2	-	39.3	-
9	48.5	1.70, *m*	47.3	1.68, *m*	47.2	1.70, *m*
10	37.0	-	37.1	-	37.2	-
11	24.2	1.99 (2H), *m*	24.0	1.99 (2H), *m*	24.4	1.99 (2H), *m*
12	129.4	5.33, t (3.5)	128.0	5.33, t (3.5)	128.9	5.32, t (3.5)
13	138.4	-	138.4	-	138.5	-
14	42.1	-	41.3	-	41.2	-
15	29.1	1.32, 1.02, m	28.9	1.32, 1.04, m	28.8	1.32, 1.02, *m*
16	26.2	2.63, 1.66, m	25.6	2.62, 1.66, m	25.8	2.63, 1.67, *m*
17	48.6	-	48.8	-	47.7	-
18	54.6	2.56, *s*	53.9	2.56, *s*	53.9	2.53, *s*
19	72.8	-	72.8	-	73.1	-
20	42.1	1.38, *m*	41.9	1.38, *m*	42.2	1.39, *m*
21	26.1	1.77 (2H), *m*	26.1	1.77 (2H), *m*	26.1	1.77 (2H), *m*
22	37.4	1.80, 1.65, *m*	37.4	1.80, 1.65, *m*	37.4	1.80, 1.67, *m*
23	27.5	1.07, *s*	27.8	1.07, *s*	27.7	1.03, *s*
24	16.8	0.80, *s*	15.9	0.86, *s*	16.8	0.87, *s*
25	16.5	0.88, *s*	15.7	0.98, *s*	15.9	0.97, *s*
26	16.3	0.96, *s*	16.2	0.79, *s*	16.6	0.79, *s*
27	24.3	1.33, *s*	23.2	1.33, *s*	23.4	1.33, *s*
28	178.5	-	178.5	-	179.0	-
29	26.8	1.22, *s*	26.1	1.22, *s*	27.4	1.22, *s*
30	16.3	0.96, d (7.0)	15.2	0.95, d (7.0)	16.3	0.95, d (7.0)

**Table 3:** ^13^C and ^1^H NMR data (*J* in Hz) of the sugar moieties of compounds **22**, **23**, and **24** (150 and 600 MHz, δ ppm, in methanol-*d*4).

	22		23		24	
	δ_C_	δ_H_ (*J* in Hz)	δ_C_	δ_H_ (*J* in Hz)	δ_C_	δ_H_ (*J* in Hz)
α- L-Ara (at C-3)				β- D -Xyl (at C-3)		α- L-Ara (at C-3)
1	106.7	4.32, *d* (7.0)	105.6	4.31, *d* (7.4)	103.5	4.56, *d* (4.0)
2	72.1	3.68, *dd* (8.0, 7.0)	74.6	3.23, *dd* (8.0, 7.0)	76.1	3.77, *dd* (8.0, 7.0)
3	74.1	3.53, *dd* (8.0, 3.0)	77.1	3.54, *dd* (8.0, 3.0)	72.9	3.75, *dd* (8.0, 3.0)
4	67.8	3.69, *m*	71.1	3.45, *m*	70.1	3.80, *m*
5	65.3	3.86, *dd*	66.2	3.88, *dd*	63.1	3.81, *dd*
		3.55, *dd*		3.57, *dd*		3.42, *dd*
						α- L-Rha (at C-2_ara_)
1					102.1	5.11, *d* (1.7)
2					72.1	3.88, *dd* (1.7, 3.4)
3					72.2	3.71, *brd*
4					73.8	3.41, *brd*
5					68.2	3.82, *brd*
6					17.9	1.23, *d* (6.2)
	β- D-Glc (at C-28)			β- D-Glc (at C-28)		β- D-Glc (at C-28)
1	95.4	5.31, *d* (7.5)	94.5	5.31, *d* (7.5)	94.8	5.30, *d* (7.5)
2	72.3	3.35, *dd* (7.5, 9.0)	72.3	3.35, *dd* (7.5, 9.0)	72.1	3.34, *dd* (7.5, 9.0)
3	77.2	3.36 *dd* (9.0, 9.0)	77.1	3.36 *dd* (9.0, 9.0)	77.2	3.45 *dd* (9.0, 9.0)
4	70.5	3.45 *dd* (9.0, 9.0)	70.1	3.45 *dd* (9.0, 9.0)	70.1	3.44 *dd* (9.0, 9.0)
5	76.7	3.52, *m*	76.8	3.52, *m*	76.5	3.51, *m*
6	68.4	3.85, *dd* (12.0, 3.5)	68.4	4.12, *dd* (12.0,3.5)	67.9	4.10, *dd* (12.0, 3.5)
		3.77, *dd* (12.0, 4.5)		3.78, *dd* (12.0,4.5)		3.76, *dd* (12.0,4.5)
	β- D-All (at C-6_Glc_)		β- D-All (at C-6_Glc_)			β- D-All (at C-6_Glc_)
1	102.1	4.70, *d* (8.0)	102.1	4.70, *d* (8.0)	102.0	4.70, *d* (8.0)
2	71.9	3.55, *dd* (8.0, 2.8)	71.9	3.55, *dd* (8.0, 2.8)	71.9	3.55, *dd* (8.0, 2.8)
3	72.3	4.08, *dd* (2.8, 2.8)	72.3	4.08, *dd* (2.8, 2.8)	72.1	4.09, *dd* (2.8, 2.8)
4	68.9	3.84, *dd* (2.8, 9.0)	68.9	3.83, *dd* (2.8, 9.0)	68.9	3.83, *dd* (2.8, 9.0)
5	76.2	3.92, *m*	76.1	3.91, *m*	75.9	3.90, *m*
6	62.1	3.85, *dd* (12.0, 3.5)	62.2	3.85, *dd* (12.0, 3.5)	62.5	3.85, *dd* (12.0, 3.5)
		3.68, *dd* (12.0, 4.5)		3.68, *dd* (12.0, 4.5)		3.68, *dd* (12.0, 4.5)

## Data Availability

The data presented in this study are available in the main article.
